# Are Some Ways of Expressing Gratitude More Beneficial Than Others? Results From a Randomized Controlled Experiment

**DOI:** 10.1007/s42761-022-00160-3

**Published:** 2022-11-07

**Authors:** Annie Regan, Lisa C. Walsh, Sonja Lyubomirsky

**Affiliations:** 1 University of California, Riverside, Riverside, USA; 2 Present Address: University of California, Los Angeles, Los Angeles, USA

**Keywords:** Gratitude, Well-being, Intervention

## Abstract

Gratitude activities have been shown to increase well-being and other positive outcomes in numerous experiments to date. The current study tested whether self-directed gratitude interventions that vary by type (i.e., social vs. nonsocial) and format (i.e., long-form letters vs. shorter lists) produce differential benefits. To that end, 958 Australian adults were assigned to one of six activities to complete each day for 1 week, including five gratitude activities that varied by type and format and an active control condition (i.e., keeping track of daily activities). Regressed change analyses revealed that, overall, long-form writing exercises (i.e., essays and letters) resulted in greater subjective well-being and other positive outcomes than lists. Indeed, those who were instructed to write social and nonsocial gratitude *lists* did not differ from controls on any outcomes. However, participants who wrote unconstrained gratitude lists—that is, those who wrote about any topics they wanted—reported greater feelings of gratitude and positive affect than did controls. Finally, relative to the other gratitude conditions, participants who wrote gratitude letters to particular individuals in their lives not only showed stronger feelings of gratitude, elevation, and other positive emotions but also reported feeling more indebted. This study demonstrates that not only does gratitude “work” to boost well-being relative to an active neutral activity, but that some forms of gratitude may be more effective than others. We hope these findings help scholars and practitioners to develop, tailor, implement, and scale future gratitude-based interventions.

**Supplementary Information:**

The online version contains supplementary material available at 10.1007/s42761-022-00160-3.

As a psychological construct, gratitude involves acknowledging a benefit received from a benefactor or an external source (Emmons & McCullough, 2003). Gratitude can be conceptualized as both a fleeting emotional state (i.e., the momentary experience of thankfulness) and as a trait (i.e., the tendency to experience this state). The present research focused on the state, or short-term, experience of gratitude.

Scholars differ in their definitions of gratitude, with some proposing that gratitude is an inherently social process, involving recognition of a kindness or benefit conferred from another person (Emmons, 2004). Others have suggested that individuals experience different types of gratitude, depending on whether the feeling is triggered by a specific benefit versus general appreciation, or whether one is grateful *to* another person versus *for* the circumstances of one’s life (Ahrens & Forbes, 2014; Lambert et al., 2009; Steindl-Rast, 2004). A goal of the current study is to better understand the complexities of experienced gratitude, whether in response to a specific kindness extended by an individual or as a global feeling of appreciation for one’s life fortunes.

## Gratitude as an Interpersonal Process

Although individuals can feel grateful for their life circumstances, life events, or their material possessions, gratitude has unique social implications—namely, to develop, maintain, and strengthen interpersonal relationships (Algoe, 2012). Indeed, numerous studies have revealed the relational benefits of gratitude, including greater relationship satisfaction and relationship maintenance behavior (Gordon et al., 2012; Kubacka et al., 2011). These relational benefits may, however, come at a cost, as gratitude is sometimes experienced as a mixed emotional state (Layous et al., 2017). For example, participants reported more indebtedness, guilt, and shame when they wrote a gratitude letter to some*one* important versus about some*thing* important in their lives (Oishi et al., 2019). The present study investigates the potential for gratitude to elicit both positive and negative feelings.

## Gratitude Interventions

In addition to its interpersonal benefits, gratitude is associated with numerous positive outcomes. Correlational research demonstrates robust associations between gratitude and positive psychological outcomes, and experimental work shows that gratitude interventions boost both the affective and cognitive components of subjective well-being (Armenta et al., 2020; Davis et al., 2016; Rash et al., 2011; Sheldon & Yu, 2021) and reduce depression and anxiety (meta-analytic *g* = −0.23; Cregg & Cheavens, 2021). Gratitude interventions have been shown to impact beneficial psychological outcomes beyond subjective well-being, including feelings of connectedness with others, elevation, and self-improvement motivation (Armenta et al., 2020; Layous et al., 2017; Walsh, Regan, & Lyubomirsky, 2022).

Notably, experimental manipulations of gratitude draw on a variety of gratitude activities (e.g., prompting participants to express gratitude through letters, lists, or verbally), and more research is needed to understand how features of these activities impact their efficacy (Jans-Beken et al., 2019). Researchers have begun to investigate the nuanced differences between methods of gratitude expression, but more work is needed to understand how specific features of these activities impact well-being and other positive outcomes (Kaczmarek et al., 2015; Sheldon & Yu, 2021; Walsh, Regan, Twenge et al., 2022). Because gratitude activities may differ in format (e.g., writing a gratitude letter or a gratitude list) and content (e.g., expressing gratitude to a specific person or about the conditions of one’s life), they may differentially impact psychological functioning and well-being. Participants are likely to write fewer words when asked to list blessings, for example, than when asked to write a gratitude letter to a specific other. The open-ended format of a gratitude letter or essay may prompt participants to write more expressively, a process that has been associated with positive outcomes and the reduction of depressive symptoms in previous research (Booker & Dunsmore, 2017; Gortner et al., 2006; Toepfer & Walker, 2009). Conversely, expressing gratitude to a specific benefactor in letter form could also be relatively more difficult or uncomfortable for participants, potentially leading to feelings of indebtedness and other socially-relevant negative emotions. An aim of the present research is to understand whether the format of gratitude interventions leads to different psychological outcomes.

## Present Study

The present study contrasted gratitude activities varying by format (letters vs. lists) and target (social or gratitude “to” vs. nonsocial or gratitude “for”). Varying features of gratitude activities allowed for direct comparisons to identify the elements that are most relevant to well-being outcomes. To that end, we randomly assigned participants to engage in one of four gratitude writing activities varying by format and target: (1) social gratitude letters, (2) nonsocial gratitude letters, (3) social gratitude lists, and (4) nonsocial gratitude lists. Participants who wrote gratitude letters did so privately—that is, they completed the exercise as part of an online survey assessment and were *not* instructed to deliver them. Instructing participants to deliver their gratitude letters (or lists) would have not only created a confound (i.e., sharing the letters in addition to writing them), but may also have caused the intervention to backfire for some participants (Fritz & Lyubomirsky, 2018; Ruini & Mortara, 2022).

In addition to testing theory-driven research questions, the present study also had a practical aim—namely, to contrast multiple gratitude intervention(s) to inform evidence-based recommendations, as gratitude interventions are already used in a variety of applied contexts, including schools (Renshaw & Olinger Steeves, 2016) and therapeutic settings (Emmons & Stern, 2013). In service of this practical aim, we included a comparison condition similar to the “counting blessings” (or “gratitude journal”) intervention that is commonly used in research and practice. Participants in this condition were not constrained to write about either people or things for which they are grateful, but rather were able to freely list whatever came to mind as a source of gratitude.

Finally, despite accumulating evidence for the benefits of gratitude, questions remain about the effect sizes and practical implications of gratitude interventions (Davis et al., 2016). As is the case with other self-administered well-being interventions, gratitude activities tend to yield small effects (Dickens, 2017). Given the relatively “low-touch” nature of these interventions, small effect sizes are to be expected, and may accumulate over time (Funder & Ozer, 2019). Although the main focus of the present research was to understand the implications of varying the target and format of gratitude interventions, we also included an active control condition to establish the overall efficacy of gratitude interventions relative to a neutral activity. Comparing gratitude activities to a neutral activity allowed us to rule out whether their effects are due to a placebo effect of simply engaging in a new activity and tracking one’s feelings over time.

In sum, the current study was designed to address four research questions:

1. Do gratitude activities “work”? We hypothesized that each of the gratitude conditions would lead to greater well-being benefits than an active control condition.
2. Do social gratitude activities outperform nonsocial ones? We hypothesized that social gratitude conditions would lead to greater well-being benefits than nonsocial gratitude conditions.
3. Does gratitude expressed via longer writing formats (letters or essays) have stronger effects than gratitude expressed via shorter lists? We hypothesized that gratitude letters/essays would yield greater well-being benefits than gratitude lists.
4. Do social gratitude letters outperform other gratitude exercises? Given our predictions about the most efficacious target (social over nonsocial) and format (letter over list), we hypothesized that those assigned to express gratitude to specific people would experience the strongest well-being outcomes of all conditions.

## Method

### Participants

Australian adults (*N* = 958; 52.2% female) were recruited from Pureprofile, an online panel company. All participants, including those in the active control condition, were told that they may be asked to participate in “positive activities” but were not told that the activities would improve their well-being.^1^ Most (87%) were White, 10.5% were Black, and the remainder were other ethnicities (4.8%). Participants ranged in age from 18 to 89 (*M* = 47.8, *SD* = 16.9), with the majority (65.6%) having at least one child (*M =* 2.34, *SD* = 1.18; range = 0 to 8 children).

### Procedure

**Fig. 1:**
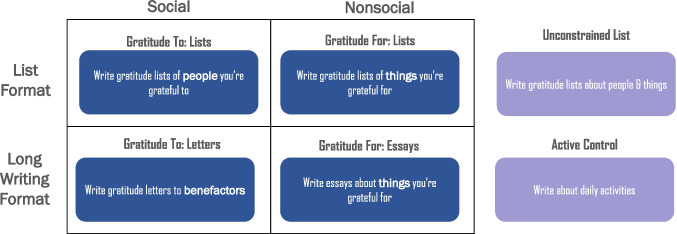
Study design

Participants were randomly assigned to one of six conditions, including four conditions that varied by type of gratitude (social/“gratitude to” vs. nonsocial/“gratitude for”) and format (list format vs. letters or essays): *social gratitude letters, nonsocial gratitude letters, social gratitude lists, nonsocial gratitude lists.* The other two comparison conditions comprised *unconstrained gratitude lists* and *active control* (see Fig. 1 for an overview of the study design).

Participants in the *social gratitude letter* condition (*n* = 167) were instructed to write gratitude letters to benefactors, while those in the *nonsocial gratitude letter* (*n* = 154) condition were instructed to write essays about things for which they are grateful, excluding people. Similarly, those assigned to the *social gratitude list* condition (*n* = 160) were instructed to write lists of people to whom they are grateful, while those in the *nonsocial gratitude list* (*n* = 170) condition were instructed to write lists of things for which they are grateful, excluding people. Participants assigned to the *unconstrained list* condition (*n* = 158) were prompted to write lists of people *or* things for which they are grateful. Finally, participants assigned to the *active control condition* (*n* = 149) were instructed to write about their daily activities. This study was approved by our university’s institutional review board.

The study took place over the course of 15 days, and all surveys were delivered via online Qualtrics surveys. After completing a pre-test survey (T_1_), participants were instructed to complete their assigned writing exercise each day for 1 week (T_2_–T_7_). Participants completed their daily writing exercise at the end of each daily survey, in an open-ended text box. Because a goal of the present study was to compare gratitude activities that varied by format (i.e., letters vs. lists), we chose to administer the activities multiple times across a relatively short time period to avoid burdening participants. That is, although the gratitude list activities may have been more effective if administered daily for multiple weeks, the longer letter/essay-writing activities may have become burdensome or repetitive. Participants completed a post-test survey at the end of the first week (T_8_), and a follow-up survey 1 week later (T_9_).

### Measures

Measures included in the present analyses are listed below. Participants completed a longer battery of measures in this study, but space precludes us from including all measures in this report.

#### Gratitude

Participants completed the Gratitude Questionnaire—Six Item Form (GQ-6; McCullough et al., 2002) and the emotion subscale of the Multi-Component Gratitude Measure (MCGM; Morgan et al., 2017) during pre-test (T_1_), post-test (T_8_), and follow-up (T_9_) assessments. The GQ-6 includes items such as “Lately I notice that I have much in life to be thankful for.” Like the GQ-6, the 6-item emotion subscale of the MCGM measures feelings of gratitude, but also includes specific items that capture both social (“There are so many people that I feel grateful for”) and nonsocial (“There are many things that I am grateful for”) gratitude. Items from both the GQ-6 and MCGM were rated on 7-point Likert scales, from 1 (*strongly disagree*) to 7 (*strongly agree*)*.* Both measures of gratitude demonstrated good reliability, with ωs ranging across all timepoints from .86 to .88 for the GQ-6 and from .93 to .94 for the MCGM. The GQ-6 and MCGM were originally included with the intent of creating a composite gratitude measure with subscales representing social and nonsocial gratitude. An exploratory factor analysis, however, did not support this approach. Given the results of this factor analysis and a correlation of .82 (*p* < .001) between these measures at pre-test, the GQ-6 and MCGM were combined into a single composite measure of state gratitude.

#### Life Satisfaction

Participants completed the 5-item Satisfaction With Life Scale (SWLS; Diener et al., 1985) at T_1_, T_8_, and T_9_, and a single item (“I am satisfied with my life”) at T_2_–T_7_ (1 = *strongly disagree*; 7 *= strongly agree*)*.* The reliability of the SWLS ranged from ω = .92 to .93 across all timepoints.

#### Affect

A modified 19-item version of the Affect Adjective Scale (AAS; Diener & Emmons, 1984) was administered at all nine timepoints. The AAS measures the extent to which participants felt positive (e.g., pleased) and negative (e.g., frustrated) affect, and, notably, includes both high-arousal (e.g., joy) and low-arousal (e.g., peaceful/serene) emotions. All items were rated on a scale from 1 (*not at all)* to 7 (*extremely*). The reliability of the composites ranged from ω = .94 to .96 for positive affect and from ω = .89 to .91 for negative affect across all timepoints.

Based on previous research on the proximal experience of gratitude interventions, we also added socially relevant negative emotions to the standard AAS, including indebted, embarrassed, uncomfortable, guilty, and ashamed (Layous et al., 2017). The present analysis focuses on the single-item measure of indebtedness in light of prior research showing that gratitude interventions specifically evoke feelings of indebtedness (Layous et al., 2017; Oishi et al., 2019).

#### Psychological Needs

Participants completed a modified version of the Balanced Measure of Psychological needs (BMPN; Sheldon & Hilpert, 2012) at all nine timepoints. Each psychological need was assessed by three items rated on a scale from 1 (*not at all*) to 7 (*very much*). Sample items include “I feel very capable in what I do” (competence), “I feel free to do things my own way” (autonomy), and “I feel close and connected with other people who are important to me” (connectedness). Reliability ranged from: ω = .83 to .95 for the competence items; ω = .86 to .94 for the autonomy items; and ω = .91 to .96 for the connectedness items.

#### Elevation

At all nine timepoints, participants completed a 6-item measure of elevation (Schnall et al., 2010), rating the extent to which they experienced feelings of elevation (e.g., “a warm feeling in your chest” and “uplifted”; 1 = *do not feel at all*; 7 = *feel very strongly*) over the past seven days (T_1,_ T_8,_ T_9_) or “right now” (T_2_–T_7_). The reliability for elevation ranged from ω = .89 to .94 across all timepoints.

### Analytic Approach

Although participants completed measures at all nine timepoints, the present analysis focused on testing condition differences only at post-test and follow-up. To test the hypothesized differences in outcomes by condition, we used regressed (i.e., residualized) change models to predict post-test and follow-up scores from condition, while holding pre-test scores constant. This analytic approach allowed for the comparison between conditions at post-test and follow-up while statistically accounting for participants’ pre-test levels of each outcome measure. Further, this approach was better suited for data with unequal sample sizes per condition than other methods (e.g., repeated-measures ANOVA). All regression coefficients were converted to partial correlations for ease of interpretation and comparability between models. We applied the Benjamini-Hochberg procedure to control the false discovery rate given the large number of comparisons in our analyses (Benjamini & Hochberg, 1995).

To determine whether attrition in our sample was random or systematic, we conducted logistic regression analyses predicting missingness at post-test and follow-up from condition. These analyses indicated that missingness differed slightly by condition, such that those in the social letter and social list writing conditions were more likely than those in the control condition to have missing data at post-test and follow-up, and those in the nonsocial essay condition were more likely than those in the control group to have missing data at follow-up (see Table 1 for means, standard deviations, and sample sizes by condition at each timepoint). To account for missing data and unequal sample sizes due to attrition, all models were estimated using a structural equation modeling approach to employ full information maximum likelihood (FIML) in the estimation of model parameters. FIML has been shown to produce less biased parameter estimates than other methods of handling missing data, such as multiple imputation and pairwise and listwise deletion (Enders & Bandalos, 2001). All models were estimated using the lavaan R package (Rosseel, 2012).

**Table 1:** Means and standard deviations for each outcome

	*N*	Autonomy	Competence	Connectedness	Elevation	Gratitude	Indebtedness	Positive affect	Negative affect	Life satisfaction
Active control										
Pre-test	149	5.01 (1.36)	4.70 (1.35)	4.95 (1.50)	4.25 (1.26)	5.21 (1.0)	3.02 (1.58)	4.15 (1.19)	2.78 (1.24)	4.25 (1.36)
Post-test	110	5.24 (1.40)	5.14 (1.31)	5.08 (1.49)	4.08 (1.60)	5.11 (1.16)	2.54 (1.74)	4.08 (1.40)	1.95 (1.06)	4.61 (1.39)
Follow-up	107	5.36 (1.38)	5.16 (1.24)	5.17 (1.56)	4.20 (1.48)	5.19 (1.17)	2.62 (1.78)	4.22 (1.37)	2.07 (1.1)	4.59 (1.33)
Nonsocial letter										
Pre-test	154	5.09 (1.22)	4.76 (1.24)	5.08 (1.32)	4.28 (1.2)	5.19 (0.94)	3.05 (1.62)	4.28 (1.2)	2.76 (1.20)	4.38 (1.44)
Post-test	100	5.59 (1.25)	5.37 (1.33)	5.54 (1.26)	4.51 (1.53)	5.55 (0.87)	2.96 (1.89)	4.54 (1.35)	1.87 (1.0)	4.96 (1.44)
Follow-up	91	5.51 (1.28)	5.27 (1.21)	5.40 (1.25)	4.54 (1.46)	5.47 (0.97)	2.85 (1.83)	4.54 (1.34)	2.06 (1.09)	4.75 (1.51)
Nonsocial list										
Pre-test	170	4.94 (1.42)	4.77 (1.49)	4.90 (1.61)	4.1 (1.38)	5.05 (1.16)	3.20 (1.71)	4.17 (1.38)	2.87 (1.34)	4.10 (1.65)
Post-test	126	5.32 (1.43)	5.18 (1.51)	5.39 (1.59)	4.08 (1.66)	5.18 (1.19)	2.78 (1.91)	4.21 (1.57)	2.08 (1.12)	4.56 (1.50)
Follow-up	115	5.41 (1.42)	5.32 (1.42)	5.37 (1.56)	4.16 (1.61)	5.26 (1.19)	2.83 (1.85)	4.22 (1.60)	2.12 (1.04)	4.52 (1.51)
Social letter										
Pre-test	167	4.93 (1.48)	4.58 (1.46)	4.77 (1.68)	4.07 (1.44)	5.11 (1.18)	3.06 (1.67)	4.22 (1.43)	2.74 (1.42)	4.23 (1.54)
Post-test	107	5.30 (1.46)	5.14 (1.49)	5.31 (1.55)	4.65 (1.59)	5.44 (1.11)	3.93 (2.0)	4.51 (1.55)	2.06 (1.27)	4.70 (1.65)
Follow-up	99	5.21 (1.55)	5.01 (1.58)	5.24 (1.57)	4.42 (1.6)	5.40 (1.17)	3.41 (2.0)	4.39 (1.56)	2.17 (1.3)	4.65 (1.73)
Social list										
Pre-test	160	5.08 (1.26)	4.63 (1.35)	5.03 (1.38)	4.27 (1.29)	5.04 (1.11)	2.99 (1.62)	4.22 (1.29)	2.79 (1.32)	4.47 (1.44)
Post-test	99	5.44 (1.23)	5.14 (1.29)	5.37 (1.38)	4.17 (1.58)	5.20 (1.15)	2.81 (1.82)	4.28 (1.38)	1.97 (1.05)	4.71 (1.50)
Follow-up	93	5.47 (1.19)	5.19 (1.22)	5.25 (1.51)	4.27 (1.52)	5.23 (1.17)	2.98 (1.84)	4.30 (1.36)	2.01 (1.06)	4.73 (1.51)
Unconstrained list										
Pre-test	158	5.25 (1.30)	4.84 (1.30)	5.24 (1.43)	4.27 (1.37)	5.25 (1.03)	2.85 (1.65)	4.45 (1.34)	2.54 (1.23)	4.47 (1.55)
Post-test	118	5.64 (1.29)	5.53 (1.18)	5.60 (1.25)	4.37 (1.56)	5.52 (1.01)	2.68 (1.89)	4.68 (1.46)	1.81 (1.04)	5.05 (1.46)
Follow-up	115	5.56 (1.25)	5.39 (1.28)	5.59 (1.32)	4.36 (1.59)	5.45 (0.96)	2.67 (1.98)	4.52 (1.48)	1.94 (1.12)	4.96 (1.51)

Finally, to contextualize our results, we conducted additional analyses to determine whether conditions differed in terms of the average number of words written by participants across timepoints. A one-way ANOVA revealed that conditions significantly differed in terms of their average word count across the intervention period, *F*(5, 592) = 22.94, *p* < .001; average word count was log-transformed. A post hoc Tukey test showed that the control, nonsocial letter, and social letter conditions each differed significantly from the unconstrained, nonsocial, and social list conditions (all *p* values < .001) but did not differ from each other in terms of average words written across all timepoints (all *p* values >.05).

## Results

### Are Gratitude Exercises Effective Relative to an Active Control Activity?

First, we ran a series of regressed change models with condition dummy-coded such that the control condition served as the reference group against all gratitude conditions combined (see Table 2 for partial *r* coefficients). Overall, participants who completed gratitude activities reported greater feelings of gratitude, indebtedness, connectedness, and elevation compared to the active control group at post-test (see Table S1 in the Supplementary information). These differences were not sustained at follow-up, with the exception of gratitude. The post-test differences in feelings of connectedness and follow-up differences in feelings of gratitude between the active control group and gratitude conditions became marginally significant after applying the Benjamini-Hochberg procedure. The post-test difference in feelings of elevation was no longer statistically significant after this procedure.

**Table 2:** Partial correlations for key comparisons

	Control vs. All Gratitude	Control vs. NS Letter	Control vs. S List	Control vs. NS List	Control vs. UC List	S Letter vs. Control	S Letter vs. NS Letter	S Letter vs. S List	S Letter vs. NS List	S Letter vs. UC List	Letters vs. Lists	To vs. For
Autonomy	.05	.07	.01	.02	.04	.04	−.03	.02	.02	.00	.07	−.02
Competence	.03	.04	.00	−.01	.05	.02	−.02	.03	.04	−.03	.07	.00
Connectedness	.07	.07	−.03	.05	.04	.08*	.00	.06	.03	.04	.07	−.02
Elevation	.06	.06	.01	.01	.05	.15**	.08**	.16**^†^	.14**	.10*	.18**^†^	.06
Gratitude	.12**	.12**	.01	.04	.12**	.14**^†^	.02	.10**	.11**	.03	.16**	.02
Indebtedness	.09**	.06	.04	.04	.02	.19**^†^	.12**	.15**	.16**	.17**^†^	.15**	.11*
Negative affect	.04	.01	.00	.05	.03	.02	.01	.00	−.03	.00	−.04	−.02
Positive affect	.06	.06	.04	.01	.08*	.10**	.04	.12**^†^	.10*	.02	.14**^†^	.02
Life satisfaction	.02	.04	−.02	−.01	.05	.04	−.01	.08*	.04	−.01	.11**	−.03

*p < .05, **p < .01. *To*, gratitude to; *For*, gratitude for; *S*, social; *NS*, nonsocial; *UC*, unconstrained. † signifies statistically significant effects at follow-up (T_9_). Statistical significance based on Benjamini-Hochberg corrected *p*-values. See supplementary tables (S1–S13) for regression coefficients, confidence intervals, and exact uncorrected and corrected *p*-values

Second, to better understand the effect of specific gratitude activities, we unpacked these results by running a series of regressed change models with condition dummy-coded such that the control condition served as the reference group against each of the other conditions. Participants in the social letter, nonsocial letter, and unconstrained list conditions significantly differed from those in the control condition at post-test (see Tables S2, S3, S4, S5, S6 in the Supplementary information). Specifically, those who wrote social gratitude letters reported feeling greater indebtedness, elevation, gratitude, positive affect, and connectedness than those in the control condition at post-test. Those who wrote nonsocial gratitude essays reported feeling higher levels of gratitude, autonomy, connectedness, and indebtedness at post-test than controls. The differences in feelings of autonomy and connectedness between the nonsocial gratitude essay and active control conditions became marginally significant after applying the Benjamini-Hochberg procedure, and the difference in feelings of indebtedness was no longer statistically significant. Participants in the unconstrained list condition reported feeling more gratitude and positive affect than controls. Participants who completed gratitude activities did not differ significantly from those in the control condition in their reported feelings of competence, negative affect, and life satisfaction.

Most condition differences mentioned above did not hold from pre-test to follow-up. The exceptions were that, compared to controls, individuals who wrote social gratitude letters reported feeling more grateful and indebted at follow-up. Individuals who wrote unconstrained gratitude lists also reported higher levels of gratitude than those who tracked their daily activities at follow-up, although this difference was no longer significant after applying the Benjamini-Hochberg procedure. Notably, those in the social and nonsocial list writing conditions did not significantly differ in any outcomes compared to those in the control condition at both post-test and follow-up.

### Are Social Gratitude Exercises More Effective Than Nonsocial Ones?

We then conducted regressed change analyses to determine whether those in the social conditions differed from those in the nonsocial conditions (excluding the unconstrained list and control conditions). Contrary to our second hypothesis, with one exception, the two social conditions did not significantly differ from the two nonsocial conditions from pre-test to post-test or follow-up (see Table S7 in Supplementary information). However, participants in the social gratitude conditions (letters and lists) reported greater feelings of indebtedness at post-test relative to those who engaged in nonsocial gratitude activities.

### Are Gratitude Letters and Essays More Effective Than Gratitude Lists?

Third, we compared the two long-form writing activities to the two gratitude list conditions to determine whether the format of gratitude interventions predicts changes in well-being and other psychological outcomes at post-test. Condition was dummy coded such that the social and nonsocial list writing activities served as a reference group for all comparisons (see Table S8 in the Supplementary information). Compared to those in the two list writing conditions, participants who wrote either social or nonsocial gratitude letters/essays reported feeling more elevation, gratitude, indebtedness, positive affect, and life satisfaction at post-test. Those in the long form writing conditions also reported feeling higher levels of elevation and positive affect at the follow-up assessment 1 week later. No statistically significant differences emerged between these conditions in psychological need satisfaction (autonomy, competence, and connectedness) or negative affect.

### Are Social Gratitude Letters More Effective Than Other Gratitude Exercises?

In light of our fourth hypothesis—namely, that the social gratitude letter would be the most effective activity—we conducted a series of regressed change models with condition dummy-coded, such that the social letter condition served as the reference group against each of the other conditions.^2^ These pairwise comparisons support the general pattern of results described previously (see Tables S9, S10, S11, S12 in the Supplementary information). Those in the social letter writing condition reported greater feelings of elevation, positive affect, gratitude, and life satisfaction relative to those in the social list condition. Participants who wrote social gratitude letters also reported experiencing more elevation, gratitude, positive affect relative to those in the nonsocial list condition. Compared to those in the nonsocial gratitude essay and unconstrained list conditions, participants who wrote social gratitude letters reported higher levels of elevation. Despite the apparent well-being benefits of writing social gratitude letters, this activity also led participants to report stronger feelings of indebtedness than each of the other gratitude conditions at post-test.

Although many of these effects were not maintained in the week following the intervention period, the results of our follow-up analyses indicated that writing social gratitude letters may have well-being benefits that are more robust relative to writing a social gratitude list. Those in the social letter writing condition reported feeling more elevation, positive affect, gratitude, connectedness, and life satisfaction at follow-up than those in the social list writing condition. The follow-up difference in feelings of gratitude between participants who wrote social gratitude letters relative to those who wrote social lists became marginally significant after applying the Benjamini-Hochberg procedure, and the follow-up differences in feelings of connectedness and life satisfaction were no longer significant. Participants’ feelings of indebtedness endured from post-test to follow-up relative to those who wrote unconstrained gratitude lists. Results of additional regressed change analyses comparing the social letter to other gratitude conditions (combined) are included in the Supplementary information (Table S13).

## Discussion

Corroborating earlier work, this study provides evidence for the efficacy of gratitude interventions relative to a neutral exercise, while also revealing nuanced differences between specific gratitude activities. As expected, participants who wrote gratitude letters or essays reported greater benefits at post-test than those who kept track of their daily activities. Surprisingly, participants who wrote social and nonsocial gratitude *lists* not only failed to differ from one another, but they performed no better than those who listed their daily activities. The long form gratitude writing activities, however, largely outperformed list writing activities in terms of well-being outcomes at post-test (i.e., greater positive affect and life satisfaction) and, to a lesser extent, at follow-up. Finally, writing social gratitude letters led to greater benefits at post-test compared to writing social or nonsocial lists. Those who wrote social gratitude letters also reported greater feelings of elevation and indebtedness at post-test than those who wrote nonsocial essays and unconstrained lists (but failed to differ in other outcomes). This pattern of results suggests that feelings of elevation and indebtedness may be unique to socially relevant expressions of gratitude (i.e., gratitude to a specific benefactor). Further, our results suggest that this increase in feelings of elevation was not due to a halo effect of greater feelings of gratitude overall, as the social letter condition was the only group that significantly differed from the active control condition for this outcome.

Notably, very few of the significant differences between conditions were sustained at follow-up, and these differences were further attenuated after correcting for multiple comparisons. These small and short-lived effects at follow-up may suggest that gratitude exercises should be completed consistently to reap their benefits over time. Although few differences between conditions reached statistical significance, our results tentatively suggest that longer-form gratitude activities (i.e., gratitude letters and essays) have slightly more durable effects than list-writing activities. Given these marginal results, future research could investigate whether administering a gratitude letter-writing intervention across several weeks would be more beneficial than administering a list-writing intervention. Intervention dosage—a moderator we did not manipulate in the present research—may be especially relevant when comparing these activities over a longer time period, as participants may feel more burdened or fatigued by repeating a letter-writing activity multiple times per week for several weeks. That is, rather than manipulate the *target* and format of gratitude activities, future investigators could examine the optimal *dosage* and format of these activities to maximize the durability of their effects (cf. Lyubomirsky & Layous, 2013).

The present research has several limitations. First, participants only engaged in gratitude activities over a single week. Future research could examine the effects of practicing different types of gratitude activities for longer periods. Next, a stronger measure of indebtedness is needed in future work when comparing social and nonsocial gratitude, as the present study relied on a single-item measure. Future research could also measure other socially relevant positive emotions to further disentangle the unique subjective experience of expressing different “types” of gratitude (i.e., social vs. nonsocial). We also acknowledge that although our sample was more representative with regard to age and gender than the primarily college student samples used in previous gratitude interventions, our participants were mostly white, Australian adults (i.e., sampled from a Western, Educated, Industrialized, Rich, Democratic, or “WEIRD” population; Henrich et al., 2010), which significantly limits the generalizability of our findings. Thus, these effects should be replicated cross-culturally, and in a more ethnically diverse sample. Finally, an examination of moderators and mechanisms underlying the relationship between gratitude activities and psychological outcomes was beyond the scope of the current paper.

In sum, the results of this study hold implications not only for well-being and gratitude scholars, but for laypeople and practitioners. First, we found that writing a gratitude *letter* appears to be a more psychologically rich experience than simply writing a *list* of people or things for which one is grateful. Furthermore, this work adds to the body of evidence that gratitude—especially when expressed in narrative form—can be leveraged to enhance subjective well-being and other positive psychological outcomes. At the same time, following previous research, our study highlights that gratitude can be a mixed emotional experience. As such, we suggest that gratitude interventions be implemented with wisdom and care—that is, recognizing that reflecting on benefits received from others may lead a person to feel both uplifted and indebted by the same gift.

## Supplementary Information

ESM 1 (DOCX 82 kb)

## Footnotes

1 The study consent form included the following language: “The goal of this study is to examine positive activities. Some of the questions will concern your personality, daily habits, thoughts, and emotions.”

2 Because the social letter condition was dummy coded to serve as the reference group for these comparisons, effect sizes for outcomes where the social letter outperformed the other conditions are negative. These effect sizes have been multiplied by −1 to convey results in terms of the social letter’s efficacy in relation to other conditions.

## Additional Information

### Data Availability

Data, materials, and R code will be available on the Open Science Foundation at https://osf.io/rp34a/?view_only=9857064e8d2544c69889f9908ce14c12.
