# Supplementary material for: Are Some Ways of Expressing Gratitude More Beneficial Than Others? Results From a Randomized Controlled Experiment
Source: Affect Sci. 2022 Nov 7;4(1):72–81. doi: 10.1007/s42761-022-00160-3 (PMC10104980; doi:10.1007/s42761-022-00160-3)
Supplement: Supplementary file 1 — (DOCX 82 kb) [file 42761_2022_160_MOESM1_ESM.docx]

**Are Some Ways of Expressing Gratitude More Beneficial Than Others?: Results From a Randomized Controlled Experiment**

Annie Regan, Lisa C. Walsh, and Sonja Lyubomirsky

Correspondence: Annie Regan (arega008@ucr.edu)

**Supplemental Materials**

**Condition Instructions**

**Nonsocial Letter Instructions**

There are many things in our lives, both large and small, that we might be grateful about. Please take a moment to think back over the past several years of your life and remember the things (excluding people) for which you are extremely grateful. Think of the things —events, experiences, opportunities, activities, situations, and so on — that have been especially wonderful for you. For example, you may feel grateful for your favorite song, traveling to a faraway place, or the opportunity to go to college.

Now, write about one of these things in detail. Remember: We are interested in the things, opportunities, or events you’re grateful for, not people you’re grateful to.

Use the instructions below to help guide you through this process:

Use whatever essay writing format you like.

Do not worry about perfect grammar and spelling.

Describe in specific terms the thing that you are grateful for and how it has affected your life. Describe what you are doing now and how you remember the positive thing, event, or experience.

Remember: Anything you write will remain strictly confidential. In fact, we do not want you to show what you write to anyone else. For the purposes of this study, this is a private document in which you can express your gratitude freely without intent to share it with anyone. Should an experimenter read this entry in the future, it will be identifiable only by a subject number and not by a name.

**Nonsocial List Instructions**

There are many things in our lives, both large and small, that we might be grateful about. Please take a moment to think back over the past several years of your life and remember the things (excluding people) for which you are extremely grateful. Think of the things —events, experiences, opportunities, activities, situations, and so on — that have been especially wonderful for you. For example, you may feel grateful for your favorite song, traveling to a faraway place, or the opportunity to go to college.

Now, in the textbox provided below, please list up to five things in your life that you are grateful or thankful for. Remember: We are interested in the things, opportunities, or events you’re grateful for, not people you’re grateful to.

Use the instructions below to help guide you through this process:

Use whatever list format you like (bulleted, numbered, etc.).
Do not worry about perfect grammar and spelling.

Remember: Anything you write will remain strictly confidential. In fact, we do not want you to show what you write to anyone else. For the purposes of this study, this is a private document in which you can express your gratitude freely without intent to share it with anyone. Should an experimenter read this entry in the future, it will be identifiable only by a subject number and not by a name.

**Social Letter Instructions**

There are many people in our lives, both close and distant, that we might be grateful about. Please take a moment to think back over the past several years of your life and remember an instance when someone did a kind act (or acts) for you for which you are extremely grateful. Think of the people – parents, children, spouses/partners, relatives, friends, neighbors, teachers, employers, and so on – who have been especially generous and thoughtful towards you. For example, you may feel grateful for a friend who gave you a compliment or a parent who helped you.

 Now, write a letter to one of these individuals.

Use the instructions below to help guide you through this process:

Use whatever letter format you like, but remember to write as though you are directly addressing the individual you are grateful to. If it is helpful to head the letter “Dear so- and-so,” or end with “Sincerely, XXX,” feel free to do so.

Do not worry about perfect grammar and spelling.

Describe in specific terms the kind act this person bestowed upon you and how the kind act affected your life.

Describe what you are doing now and how you often remember their efforts.

Remember: Anything you write will remain strictly confidential. In fact, we do not want you to show what you write to anyone else. For the purposes of this study, this is a private document in which you can express your gratitude freely without intent to deliver it to anyone. Should an experimenter read this entry in the future, it will be identifiable only by a subject number and not by a name.

**Social List Instructions**

There are many people in our lives, both close and distant, that we might be grateful about. Please take a moment to think back over the past several years of your life and remember the individuals for which you are extremely grateful. Think of the people – parents, children, spouses/partners, relatives, friends, neighbors, teachers, employers, and so on – who have been especially generous and thoughtful towards you. For example, you may feel grateful for a friend who gave you a compliment or a parent who helped you.

Now, in the textbox provided below, please list up to five people in your life that you are grateful or thankful for. Remember: We are interested in the people you’re grateful to, not the things, opportunities, or events you’re grateful for.

Use the instructions below to help guide you through this process:

Use whatever list format you like (bulleted, numbered, etc.). Do not worry about perfect grammar and spelling.

Remember: Anything you write will remain strictly confidential. In fact, we do not want you to show what you write to anyone else. For the purposes of this study, this is a private document in which you can express your gratitude freely without intent to share it with anyone. Should an experimenter read this entry in the future, it will be identifiable only by a subject number and not by a name.

**Unconstrained List Instructions**

There are many things in our lives, both large and small, that we might be grateful about. Think back over the past several years of your life and write down in the textboxes below up to five things in your life that you are grateful or thankful for.

We are interested in the things, people, opportunities, events, activities, situations, and so on that you're grateful about.

**Active Control Instructions**

Please take a moment to think about what you did yesterday. That is, create a mental outline of what you did during that time. Now please write these activities out in a list format.

Use the instructions below to help guide you through this process:

Use whatever writing format you like.

Try to leave out emotions, feelings, or opinions pertaining to your plans.

Focus on exactly what you did.

Do not worry about perfect grammar and spelling.

Remember: Anything you write will remain strictly confidential. In fact, we do not want you to show what you write to anyone else. For the purposes of this study, this is a private document in which you can write freely without intent to share it with anyone. Should an experimenter read this entry in the future, it will be identifiable only by a subject number and not by a name.

| **Table S1.** |  |  |  |  |  |  |  |
| --- | --- | --- | --- | --- | --- | --- | --- |
| *Residualized Change Models Comparing the Effects of All Gratitude Activities (combined) and the Active Control Condition at Post-Test (T8) and Follow-Up (T9).* | | | | | | | |
| **Outcome** | ***b*** | ***SE*** | **Partial *r*** | **Partial *r* LLCI** | **Partial *r* ULCI** | ***p*** | ***p (adjusted)*** |
| Autonomy |  |  |  |  |  |  |  |
| Post-test | 0.14 | 0.10 | 0.05 | -0.02 | 0.11 | 0.16 | 0.31 |
| Follow-up | -0.01 | 0.11 | 0.00 | -0.07 | 0.06 | 0.89 | 0.94 |
| Competence |  |  |  |  |  |  |  |
| Post-test | 0.08 | 0.11 | 0.03 | -0.04 | 0.09 | 0.44 | 0.61 |
| Follow-up | 0.02 | 0.11 | 0.01 | -0.06 | 0.07 | 0.88 | 0.94 |
| Connectedness |  |  |  |  |  |  |  |
| Post-test | 0.21 | 0.10 | 0.07 | 0.00 | 0.13 | 0.04 | 0.09 |
| Follow-up | 0.04 | 0.11 | 0.01 | -0.05 | 0.08 | 0.69 | 0.79 |
| Elevation |  |  |  |  |  |  |  |
| Post-test | 0.22 | 0.11 | 0.06 | 0.00 | 0.13 | 0.05 | 0.11 |
| Follow-up | 0.08 | 0.11 | 0.02 | -0.04 | 0.09 | 0.48 | 0.63 |
| Gratitude |  |  |  |  |  |  |  |
| Post-test | 0.25 | 0.07 | 0.12 | 0.05 | 0.18 | < .001 | < .001 |
| Follow-up | 0.15 | 0.07 | 0.07 | 0.01 | 0.13 | 0.03 | 0.08 |
| Indebtedness |  |  |  |  |  |  |  |
| Post-test | 0.46 | 0.17 | 0.09 | 0.02 | 0.15 | 0.01 | 0.02 |
| Follow-up | 0.26 | 0.17 | 0.05 | -0.01 | 0.11 | 0.12 | 0.24 |
| Negative Affect |  |  |  |  |  |  |  |
| Post-test | 0.08 | 0.08 | 0.04 | -0.03 | 0.10 | 0.26 | 0.43 |
| Follow-up | 0.06 | 0.08 | 0.02 | -0.04 | 0.09 | 0.46 | 0.62 |
| Positive Affect |  |  |  |  |  |  |  |
| Post-test | 0.17 | 0.10 | 0.06 | -0.01 | 0.12 | 0.08 | 0.17 |
| Follow-up | 0.01 | 0.10 | 0.00 | -0.06 | 0.07 | 0.92 | 0.96 |
| Life Satisfaction |  |  |  |  |  |  |  |
| Post-test | 0.05 | 0.08 | 0.02 | -0.04 | 0.08 | 0.56 | 0.70 |
| Follow-up | -0.03 | 0.09 | -0.01 | -0.07 | 0.05 | 0.77 | 0.85 |
| *Note.* Condition was dummy-coded such that all comparisons above are in relation to the control condition. | | | | | | | |

| **Table S2.** |  |  |  |  |  |  |  |
| --- | --- | --- | --- | --- | --- | --- | --- |
| *Residualized Change Models Comparing the Effects of the Nonsocial Letter vs. the Active Control Condition at Post-Test (T8) and Follow-Up (T9).* | | | | | | | |
| **Outcome** | ***b*** | ***SE*** | **Partial *r*** | **Partial *r* LLCI** | **Partial *r* ULCI** | ***p*** | ***p (adjusted)*** |
| Autonomy |  |  |  |  |  |  |  |
| Post-test | 0.30 | 0.13 | 0.07 | 0.01 | 0.13 | 0.03 | 0.07 |
| Follow-up | 0.05 | 0.14 | 0.01 | -0.05 | 0.08 | 0.72 | 0.81 |
| Competence |  |  |  |  |  |  |  |
| Post-test | 0.17 | 0.14 | 0.04 | -0.03 | 0.10 | 0.24 | 0.41 |
| Follow-up | 0.03 | 0.15 | 0.01 | -0.06 | 0.07 | 0.83 | 0.90 |
| Connectedness |  |  |  |  |  |  |  |
| Post-test | 0.30 | 0.13 | 0.07 | 0.01 | 0.13 | 0.03 | 0.06 |
| Follow-up | 0.02 | 0.14 | 0.00 | -0.06 | 0.07 | 0.90 | 0.95 |
| Elevation |  |  |  |  |  |  |  |
| Post-test | 0.27 | 0.14 | 0.06 | 0.00 | 0.12 | 0.06 | 0.13 |
| Follow-up | 0.15 | 0.15 | 0.03 | -0.03 | 0.10 | 0.32 | 0.49 |
| Gratitude |  |  |  |  |  |  |  |
| Post-test | 0.34 | 0.09 | 0.12 | 0.06 | 0.18 | < .001 | < .001 |
| Follow-up | 0.14 | 0.10 | 0.05 | -0.02 | 0.11 | 0.14 | 0.26 |
| Indebtedness |  |  |  |  |  |  |  |
| Post-test | 0.43 | 0.22 | 0.06 | 0.00 | 0.13 | 0.05 | 0.11 |
| Follow-up | 0.22 | 0.23 | 0.03 | -0.03 | 0.09 | 0.34 | 0.52 |
| Negative Affect |  |  |  |  |  |  |  |
| Post-test | 0.04 | 0.10 | 0.01 | -0.05 | 0.08 | 0.71 | 0.80 |
| Follow-up | 0.12 | 0.11 | 0.03 | -0.03 | 0.10 | 0.30 | 0.48 |
| Positive Affect |  |  |  |  |  |  |  |
| Post-test | 0.23 | 0.13 | 0.06 | -0.01 | 0.12 | 0.08 | 0.17 |
| Follow-up | 0.11 | 0.14 | 0.02 | -0.04 | 0.09 | 0.45 | 0.61 |
| Life Satisfaction |  |  |  |  |  |  |  |
| Post-test | 0.14 | 0.11 | 0.04 | -0.02 | 0.10 | 0.21 | 0.37 |
| Follow-up | -0.09 | 0.11 | -0.03 | -0.09 | 0.04 | 0.44 | 0.61 |
| *Note.* Condition was dummy-coded such that all comparisons above are in relation to the control condition. | | | | | | | |

| **Table S3.** |  |  |  |  |  |  |  |
| --- | --- | --- | --- | --- | --- | --- | --- |
| *Residualized Change Models Comparing the Effects of the Nonsocial List vs. the Active Control Condition at Post-Test (T8) and Follow-Up (T9).* | | | | | | | |
| **Outcome** | ***b*** | ***SE*** | **Partial *r*** | **Partial *r* LLCI** | **Partial *r* ULCI** | ***p*** | ***p (adjusted)*** |
| Autonomy |  |  |  |  |  |  |  |
| Post-test | 0.06 | 0.13 | 0.02 | -0.05 | 0.08 | 0.63 | 0.74 |
| Follow-up | 0.06 | 0.13 | 0.01 | -0.05 | 0.08 | 0.64 | 0.75 |
| Competence |  |  |  |  |  |  |  |
| Post-test | -0.05 | 0.13 | -0.01 | -0.08 | 0.05 | 0.71 | 0.80 |
| Follow-up | 0.07 | 0.14 | 0.02 | -0.05 | 0.08 | 0.60 | 0.72 |
| Connectedness |  |  |  |  |  |  |  |
| Post-test | 0.20 | 0.13 | 0.05 | -0.01 | 0.11 | 0.12 | 0.24 |
| Follow-up | 0.11 | 0.13 | 0.03 | -0.04 | 0.09 | 0.43 | 0.60 |
| Elevation |  |  |  |  |  |  |  |
| Post-test | 0.04 | 0.14 | 0.01 | -0.05 | 0.07 | 0.75 | 0.83 |
| Follow-up | 0.03 | 0.14 | 0.01 | -0.06 | 0.07 | 0.85 | 0.92 |
| Gratitude |  |  |  |  |  |  |  |
| Post-test | 0.11 | 0.09 | 0.04 | -0.02 | 0.10 | 0.20 | 0.37 |
| Follow-up | 0.12 | 0.09 | 0.05 | -0.02 | 0.11 | 0.16 | 0.31 |
| Indebtedness |  |  |  |  |  |  |  |
| Post-test | 0.23 | 0.21 | 0.04 | -0.03 | 0.10 | 0.26 | 0.43 |
| Follow-up | 0.20 | 0.21 | 0.03 | -0.03 | 0.09 | 0.36 | 0.54 |
| Negative Affect |  |  |  |  |  |  |  |
| Post-test | 0.16 | 0.09 | 0.05 | -0.01 | 0.12 | 0.09 | 0.19 |
| Follow-up | 0.06 | 0.10 | 0.02 | -0.04 | 0.08 | 0.54 | 0.69 |
| Positive Affect |  |  |  |  |  |  |  |
| Post-test | 0.03 | 0.12 | 0.01 | -0.06 | 0.07 | 0.84 | 0.90 |
| Follow-up | -0.06 | 0.13 | -0.01 | -0.08 | 0.05 | 0.65 | 0.75 |
| Life Satisfaction |  |  |  |  |  |  |  |
| Post-test | -0.02 | 0.10 | -0.01 | -0.07 | 0.06 | 0.83 | 0.90 |
| Follow-up | -0.03 | 0.11 | -0.01 | -0.07 | 0.05 | 0.77 | 0.85 |
| *Note.* Condition was dummy-coded such that all comparisons above are in relation to the control condition. | | | | | | | |

| **Table S4.** |  |  |  |  |  |  |  |
| --- | --- | --- | --- | --- | --- | --- | --- |
| *Residualized Change Models Comparing the Effects of the Social Letter vs. the Active Control Condition at Post-Test (T8) And Follow-Up (T9).* | | | | | | | |
| **Outcome** | ***b*** | ***SE*** | **Partial *r*** | **Partial *r* LLCI** | **Partial *r* ULCI** | ***p*** | ***p (adjusted)*** |
| Autonomy |  |  |  |  |  |  |  |
| Post-test | 0.15 | 0.13 | 0.04 | -0.03 | 0.10 | 0.25 | 0.42 |
| Follow-up | -0.08 | 0.14 | -0.02 | -0.08 | 0.04 | 0.55 | 0.69 |
| Competence |  |  |  |  |  |  |  |
| Post-test | 0.10 | 0.14 | 0.02 | -0.04 | 0.09 | 0.47 | 0.63 |
| Follow-up | -0.08 | 0.15 | -0.02 | -0.08 | 0.05 | 0.57 | 0.71 |
| Connectedness |  |  |  |  |  |  |  |
| Post-test | 0.31 | 0.13 | 0.08 | 0.01 | 0.14 | 0.02 | 0.04 |
| Follow-up | 0.14 | 0.14 | 0.03 | -0.03 | 0.10 | 0.32 | 0.49 |
| Elevation |  |  |  |  |  |  |  |
| Post-test | 0.64 | 0.14 | 0.15 | 0.08 | 0.21 | < .001 | < .001 |
| Follow-up | 0.28 | 0.15 | 0.06 | 0.00 | 0.12 | 0.06 | 0.13 |
| Gratitude |  |  |  |  |  |  |  |
| Post-test | 0.39 | 0.09 | 0.14 | 0.08 | 0.20 | < .001 | < .001 |
| Follow-up | 0.26 | 0.09 | 0.09 | 0.03 | 0.15 | 0.01 | 0.01 |
| Indebtedness |  |  |  |  |  |  |  |
| Post-test | 1.27 | 0.21 | 0.19 | 0.13 | 0.25 | < .001 | < .001 |
| Follow-up | 0.63 | 0.22 | 0.09 | 0.03 | 0.15 | 0.01 | 0.01 |
| Negative Affect |  |  |  |  |  |  |  |
| Post-test | 0.07 | 0.10 | 0.02 | -0.04 | 0.09 | 0.49 | 0.64 |
| Follow-up | 0.06 | 0.11 | 0.02 | -0.05 | 0.08 | 0.60 | 0.72 |
| Positive Affect |  |  |  |  |  |  |  |
| Post-test | 0.39 | 0.13 | 0.10 | 0.04 | 0.16 | 0.00 | 0.01 |
| Follow-up | 0.16 | 0.14 | 0.04 | -0.03 | 0.10 | 0.24 | 0.41 |
| Life Satisfaction |  |  |  |  |  |  |  |
| Post-test | 0.12 | 0.11 | 0.04 | -0.03 | 0.10 | 0.26 | 0.43 |
| Follow-up | 0.05 | 0.11 | 0.02 | -0.05 | 0.08 | 0.63 | 0.74 |
| *Note.* Condition was dummy-coded such that all comparisons above are in relation to the control condition. | | | | | | | |

| **Table S5.** |  |  |  |  |  |  |  |
| --- | --- | --- | --- | --- | --- | --- | --- |
| *Residualized Change Models Comparing the Effects of the Social List vs. the Active Control Condition at Post-Test (T8) and Follow-Up (T9).* | | | | | | | |
| **Outcome** | ***b*** | ***SE*** | **Partial *r*** | **Partial *r* LLCI** | **Partial *r* ULCI** | ***p*** | ***p (adjusted)*** |
| Autonomy |  |  |  |  |  |  |  |
| Post-test | 0.05 | 0.13 | 0.01 | -0.05 | 0.08 | 0.69 | 0.79 |
| Follow-up | -0.08 | 0.14 | -0.02 | -0.08 | 0.04 | 0.55 | 0.69 |
| Competence |  |  |  |  |  |  |  |
| Post-test | -0.01 | 0.14 | 0.00 | -0.07 | 0.06 | 0.92 | 0.96 |
| Follow-up | -0.01 | 0.15 | 0.00 | -0.07 | 0.06 | 0.96 | 0.97 |
| Connectedness |  |  |  |  |  |  |  |
| Post-test | 0.08 | 0.13 | 0.02 | -0.04 | 0.08 | 0.56 | 0.70 |
| Follow-up | -0.15 | 0.14 | -0.03 | -0.10 | 0.03 | 0.30 | 0.48 |
| Elevation |  |  |  |  |  |  |  |
| Post-test | -0.08 | 0.14 | -0.02 | -0.08 | 0.05 | 0.60 | 0.72 |
| Follow-up | -0.13 | 0.15 | -0.03 | -0.09 | 0.03 | 0.37 | 0.55 |
| Gratitude |  |  |  |  |  |  |  |
| Post-test | 0.10 | 0.09 | 0.04 | -0.03 | 0.10 | 0.26 | 0.43 |
| Follow-up | 0.05 | 0.09 | 0.02 | -0.05 | 0.08 | 0.57 | 0.71 |
| Indebtedness |  |  |  |  |  |  |  |
| Post-test | 0.25 | 0.22 | 0.04 | -0.03 | 0.10 | 0.24 | 0.41 |
| Follow-up | 0.30 | 0.23 | 0.04 | -0.02 | 0.11 | 0.19 | 0.34 |
| Negative Affect |  |  |  |  |  |  |  |
| Post-test | 0.06 | 0.10 | 0.02 | -0.04 | 0.08 | 0.53 | 0.68 |
| Follow-up | -0.01 | 0.11 | 0.00 | -0.07 | 0.06 | 0.95 | 0.97 |
| Positive Affect |  |  |  |  |  |  |  |
| Post-test | -0.09 | 0.13 | -0.02 | -0.09 | 0.04 | 0.48 | 0.63 |
| Follow-up | -0.21 | 0.14 | -0.05 | -0.11 | 0.02 | 0.14 | 0.26 |
| Life Satisfaction |  |  |  |  |  |  |  |
| Post-test | -0.15 | 0.11 | -0.04 | -0.11 | 0.02 | 0.17 | 0.31 |
| Follow-up | -0.17 | 0.11 | -0.05 | -0.11 | 0.01 | 0.13 | 0.25 |
| *Note.* Condition was dummy-coded such that all comparisons above are in relation to the control condition. | | | | | | | |

| **Table S6.** |  |  |  |  |  |  |  |
| --- | --- | --- | --- | --- | --- | --- | --- |
| *Residualized Change Models Comparing the Effects of the Unconstrained List vs. Active Control at Post-Test (T8) and Follow-Up (T9).* | | | | | | | |
| **Outcome** | ***b*** | ***SE*** | **Partial *r*** | **Partial *r* LLCI** | **Partial *r* ULCI** | ***p*** | ***p (adjusted)*** |
| Autonomy |  |  |  |  |  |  |  |
| Post-test | 0.16 | 0.13 | 0.04 | -0.02 | 0.10 | 0.21 | 0.37 |
| Follow-up | -0.03 | 0.13 | -0.01 | -0.07 | 0.06 | 0.84 | 0.91 |
| Competence |  |  |  |  |  |  |  |
| Post-test | 0.22 | 0.13 | 0.05 | -0.01 | 0.12 | 0.10 | 0.21 |
| Follow-up | 0.06 | 0.14 | 0.01 | -0.05 | 0.08 | 0.69 | 0.79 |
| Connectedness |  |  |  |  |  |  |  |
| Post-test | 0.17 | 0.13 | 0.04 | -0.02 | 0.10 | 0.20 | 0.36 |
| Follow-up | 0.06 | 0.14 | 0.02 | -0.05 | 0.08 | 0.64 | 0.75 |
| Elevation |  |  |  |  |  |  |  |
| Post-test | 0.23 | 0.14 | 0.05 | -0.01 | 0.12 | 0.10 | 0.21 |
| Follow-up | 0.08 | 0.14 | 0.02 | -0.05 | 0.08 | 0.59 | 0.72 |
| Gratitude |  |  |  |  |  |  |  |
| Post-test | 0.32 | 0.09 | 0.12 | 0.06 | 0.18 | < .001 | 0.00 |
| Follow-up | 0.18 | 0.09 | 0.06 | 0.00 | 0.13 | 0.05 | 0.11 |
| Indebtedness |  |  |  |  |  |  |  |
| Post-test | 0.15 | 0.21 | 0.02 | -0.04 | 0.09 | 0.47 | 0.63 |
| Follow-up | 0.03 | 0.21 | 0.00 | -0.06 | 0.07 | 0.89 | 0.94 |
| Negative Affect |  |  |  |  |  |  |  |
| Post-test | 0.08 | 0.10 | 0.03 | -0.04 | 0.09 | 0.41 | 0.58 |
| Follow-up | 0.07 | 0.10 | 0.02 | -0.04 | 0.09 | 0.48 | 0.63 |
| Positive Affect |  |  |  |  |  |  |  |
| Post-test | 0.31 | 0.13 | 0.08 | 0.02 | 0.14 | 0.01 | 0.03 |
| Follow-up | 0.05 | 0.13 | 0.01 | -0.05 | 0.08 | 0.71 | 0.80 |
| Life Satisfaction |  |  |  |  |  |  |  |
| Post-test | 0.15 | 0.10 | 0.05 | -0.02 | 0.11 | 0.14 | 0.27 |
| Follow-up | 0.08 | 0.11 | 0.03 | -0.04 | 0.09 | 0.44 | 0.61 |
| *Note.* Condition was dummy-coded such that all comparisons above are in relation to the control condition. | | | | | | | |

| **Table S7.** |  |  |  |  |  |  |  |
| --- | --- | --- | --- | --- | --- | --- | --- |
| *Residualized Change Models Comparing the Effects of Social vs. Nonsocial Gratitude Exercises at Post-Test (T8) and Follow-Up (T9).* | | | | | | | |
| **Outcome** | ***b*** | ***SE*** | **Partial *r*** | **Partial *r* LLCI** | **Partial *r* ULCI** | ***p*** | ***p (adjusted)*** |
| Autonomy |  |  |  |  |  |  |  |
| Post-test | -0.06 | 0.10 | -0.02 | -0.10 | 0.05 | 0.53 | 0.68 |
| Follow-up | -0.14 | 0.10 | -0.05 | -0.13 | 0.02 | 0.17 | 0.31 |
| Competence |  |  |  |  |  |  |  |
| Post-test | 0.00 | 0.10 | 0.00 | -0.08 | 0.08 | 0.98 | 0.99 |
| Follow-up | -0.10 | 0.10 | -0.04 | -0.11 | 0.04 | 0.36 | 0.54 |
| Connectedness |  |  |  |  |  |  |  |
| Post-test | -0.04 | 0.09 | -0.02 | -0.09 | 0.06 | 0.67 | 0.77 |
| Follow-up | -0.07 | 0.10 | -0.03 | -0.10 | 0.05 | 0.51 | 0.66 |
| Elevation |  |  |  |  |  |  |  |
| Post-test | 0.15 | 0.10 | 0.06 | -0.02 | 0.13 | 0.14 | 0.27 |
| Follow-up | 0.00 | 0.11 | 0.00 | -0.08 | 0.08 | 1.00 | 1.00 |
| Gratitude |  |  |  |  |  |  |  |
| Post-test | 0.04 | 0.07 | 0.02 | -0.05 | 0.10 | 0.57 | 0.71 |
| Follow-up | 0.03 | 0.07 | 0.02 | -0.06 | 0.09 | 0.68 | 0.78 |
| Indebtedness |  |  |  |  |  |  |  |
| Post-test | 0.47 | 0.16 | 0.11 | 0.04 | 0.19 | 0.00 | 0.01 |
| Follow-up | 0.27 | 0.16 | 0.06 | -0.01 | 0.14 | 0.10 | 0.21 |
| Negative Affect |  |  |  |  |  |  |  |
| Post-test | -0.04 | 0.07 | -0.02 | -0.10 | 0.05 | 0.53 | 0.68 |
| Follow-up | -0.06 | 0.08 | -0.03 | -0.11 | 0.05 | 0.43 | 0.60 |
| Positive Affect |  |  |  |  |  |  |  |
| Post-test | 0.04 | 0.09 | 0.02 | -0.06 | 0.09 | 0.65 | 0.75 |
| Follow-up | -0.03 | 0.10 | -0.01 | -0.09 | 0.07 | 0.76 | 0.84 |
| Life Satisfaction |  |  |  |  |  |  |  |
| Post-test | -0.06 | 0.08 | -0.03 | -0.11 | 0.05 | 0.45 | 0.61 |
| Follow-up | 0.00 | 0.08 | 0.00 | -0.08 | 0.08 | 1.00 | 1.00 |
| *Note.* Condition was dummy-coded such that all comparisons above are in relation to the nonsocial conditions (lists and letters). | | | | | | | |

| **Table S8.** |  |  |  |  |  |  |  |
| --- | --- | --- | --- | --- | --- | --- | --- |
| *Residualized Change Models Comparing the Effects of Long-Form Writing vs. List Writing at Post-Test (T8) and Follow-Up (T9).* | | | | | | | |
| **Outcome** | ***b*** | ***SE*** | **Partial *r*** | **Partial *r* LLCI** | **Partial *r* ULCI** | ***p*** | ***p (adjusted)*** |
| Autonomy |  |  |  |  |  |  |  |
| Post-test | 0.16 | 0.10 | 0.07 | -0.01 | 0.14 | 0.09 | 0.19 |
| Follow-up | -0.01 | 0.10 | -0.01 | -0.08 | 0.07 | 0.88 | 0.94 |
| Competence |  |  |  |  |  |  |  |
| Post-test | 0.17 | 0.10 | 0.07 | -0.01 | 0.14 | 0.09 | 0.19 |
| Follow-up | -0.06 | 0.10 | -0.02 | -0.10 | 0.05 | 0.56 | 0.70 |
| Connectedness |  |  |  |  |  |  |  |
| Post-test | 0.16 | 0.09 | 0.07 | -0.01 | 0.14 | 0.08 | 0.18 |
| Follow-up | 0.09 | 0.10 | 0.03 | -0.04 | 0.11 | 0.38 | 0.56 |
| Elevation |  |  |  |  |  |  |  |
| Post-test | 0.47 | 0.10 | 0.18 | 0.11 | 0.25 | < .001 | < .001 |
| Follow-up | 0.27 | 0.11 | 0.10 | 0.02 | 0.17 | 0.01 | 0.03 |
| Gratitude |  |  |  |  |  |  |  |
| Post-test | 0.26 | 0.06 | 0.16 | 0.08 | 0.23 | < .001 | < .001 |
| Follow-up | 0.11 | 0.07 | 0.06 | -0.01 | 0.14 | 0.10 | 0.21 |
| Indebtedness |  |  |  |  |  |  |  |
| Post-test | 0.63 | 0.16 | 0.15 | 0.08 | 0.22 | < .001 | < .001 |
| Follow-up | 0.19 | 0.16 | 0.05 | -0.03 | 0.12 | 0.23 | 0.41 |
| Negative Affect |  |  |  |  |  |  |  |
| Post-test | -0.06 | 0.07 | -0.04 | -0.11 | 0.04 | 0.35 | 0.54 |
| Follow-up | 0.05 | 0.08 | 0.03 | -0.05 | 0.10 | 0.49 | 0.64 |
| Positive Affect |  |  |  |  |  |  |  |
| Post-test | 0.34 | 0.09 | 0.14 | 0.07 | 0.21 | < .001 | < .001 |
| Follow-up | 0.26 | 0.10 | 0.10 | 0.02 | 0.18 | 0.01 | 0.03 |
| Life Satisfaction |  |  |  |  |  |  |  |
| Post-test | 0.21 | 0.08 | 0.11 | 0.03 | 0.18 | 0.01 | 0.02 |
| Follow-up | 0.08 | 0.08 | 0.04 | -0.04 | 0.11 | 0.33 | 0.51 |
| *Note.* Condition was dummy-coded such that all comparisons above are in relation to the list-writing conditions (social and nonsocial lists). | | | | | | | |

| **Table S9.** |  |  |  |  |  |  |  |
| --- | --- | --- | --- | --- | --- | --- | --- |
| *Residualized Change Models Comparing the Effects of the Social vs. Nonsocial Gratitude Letter Conditions at Post-Test (T8) and Follow-up (T9).* | | | | | | | |
| **Outcome** | ***b*** | ***SE*** | **Partial *r*** | **Partial *r* LLCI** | **Partial *r* ULCI** | ***p*** | ***p (adjusted)*** |
| Autonomy |  |  |  |  |  |  |  |
| Post-test | -0.14 | 0.14 | -0.03 | -0.10 | 0.03 | 0.29 | 0.47 |
| Follow-up | -0.13 | 0.14 | -0.03 | -0.09 | 0.03 | 0.35 | 0.54 |
| Competence |  |  |  |  |  |  |  |
| Post-test | -0.07 | 0.14 | -0.02 | -0.08 | 0.05 | 0.64 | 0.75 |
| Follow-up | -0.11 | 0.15 | -0.02 | -0.09 | 0.04 | 0.45 | 0.62 |
| Connectedness |  |  |  |  |  |  |  |
| Post-test | 0.02 | 0.13 | 0.00 | -0.06 | 0.07 | 0.90 | 0.95 |
| Follow-up | 0.12 | 0.15 | 0.03 | -0.04 | 0.09 | 0.40 | 0.58 |
| Elevation |  |  |  |  |  |  |  |
| Post-test | 0.37 | 0.15 | 0.08 | 0.02 | 0.15 | 0.01 | 0.03 |
| Follow-up | 0.13 | 0.16 | 0.03 | -0.04 | 0.09 | 0.40 | 0.57 |
| Gratitude |  |  |  |  |  |  |  |
| Post-test | 0.05 | 0.09 | 0.02 | -0.05 | 0.08 | 0.62 | 0.74 |
| Follow-up | 0.12 | 0.10 | 0.04 | -0.02 | 0.10 | 0.22 | 0.39 |
| Indebtedness |  | 0.00 |  |  |  |  |  |
| Post-test | 0.84 |  | 0.12 | 0.06 | 0.19 | < .001 | < .001 |
| Follow-up | 0.41 | 0.23 | 0.06 | -0.01 | 0.12 | 0.07 | 0.16 |
| Negative Affect |  |  |  |  |  |  |  |
| Post-test | 0.03 | 0.10 | 0.01 | -0.05 | 0.07 | 0.75 | 0.84 |
| Follow-up | -0.06 | 0.11 | -0.02 | -0.08 | 0.05 | 0.61 | 0.73 |
| Positive Affect |  |  |  |  |  |  |  |
| Post-test | 0.16 | 0.13 | 0.04 | -0.02 | 0.10 | 0.22 | 0.39 |
| Follow-up | 0.06 | 0.14 | 0.01 | -0.05 | 0.08 | 0.70 | 0.79 |
| Life Satisfaction |  |  |  |  |  |  |  |
| Post-test | -0.02 | 0.11 | -0.01 | -0.07 | 0.06 | 0.87 | 0.94 |
| Follow-up | 0.14 | 0.12 | 0.04 | -0.02 | 0.10 | 0.22 | 0.39 |
| *Note.* Condition was dummy-coded such that all comparisons above are in relation to the nonsocial letter condition. | | | | | | | |

| **Table S10.** |  |  |  |  |  |  |  |
| --- | --- | --- | --- | --- | --- | --- | --- |
| *Residualized Change Models Comparing the Effects of the Social Letter vs. Nonsocial Gratitude List Condition at Post-Test (T8) and Follow-Up (T9).* | | | | | | | |
| **Outcome** | ***b*** | ***SE*** | **Partial *r*** | **Partial *r* LLCI** | **Partial *r* ULCI** | ***p*** | ***p (adjusted)*** |
| Autonomy |  |  |  |  |  |  |  |
| Post-test | 0.09 | 0.13 | 0.02 | -0.04 | 0.09 | 0.48 | 0.63 |
| Follow-up | -0.14 | 0.14 | -0.03 | -0.10 | 0.03 | 0.29 | 0.47 |
| Competence |  |  |  |  |  |  |  |
| Post-test | 0.15 | 0.13 | 0.04 | -0.03 | 0.10 | 0.27 | 0.44 |
| Follow-up | -0.16 | 0.14 | -0.04 | -0.10 | 0.03 | 0.28 | 0.45 |
| Connectedness |  |  |  |  |  |  |  |
| Post-test | 0.12 | 0.13 | 0.03 | -0.03 | 0.09 | 0.36 | 0.54 |
| Follow-up | 0.03 | 0.14 | 0.01 | -0.06 | 0.07 | 0.81 | 0.88 |
| Elevation |  |  |  |  |  |  |  |
| Post-test | 0.60 | 0.14 | 0.14 | 0.08 | 0.20 | 0.00 | 0.00 |
| Follow-up | 0.26 | 0.15 | 0.06 | -0.01 | 0.12 | 0.08 | 0.17 |
| Gratitude |  |  |  |  |  |  |  |
| Post-test | 0.28 | 0.09 | 0.11 | 0.04 | 0.17 | 0.00 | 0.00 |
| Follow-up | 0.14 | 0.09 | 0.05 | -0.02 | 0.11 | 0.14 | 0.26 |
| Indebtedness |  |  |  |  |  |  |  |
| Post-test | 1.04 | 0.21 | 0.16 | 0.10 | 0.22 | < .001 | < .001 |
| Follow-up | 0.43 | 0.22 | 0.06 | 0.00 | 0.13 | 0.05 | 0.11 |
| Negative Affect |  |  |  |  |  |  |  |
| Post-test | -0.09 | 0.10 | -0.03 | -0.09 | 0.03 | 0.34 | 0.53 |
| Follow-up | -0.01 | 0.11 | 0.00 | -0.07 | 0.06 | 0.95 | 0.97 |
| Positive Affect |  |  |  |  |  |  |  |
| Post-test | 0.37 | 0.12 | 0.10 | 0.03 | 0.16 | 0.00 | 0.01 |
| Follow-up | 0.22 | 0.13 | 0.05 | -0.01 | 0.12 | 0.10 | 0.21 |
| Life Satisfaction |  |  |  |  |  |  |  |
| Post-test | 0.14 | 0.10 | 0.04 | -0.02 | 0.11 | 0.17 | 0.31 |
| Follow-up | 0.09 | 0.11 | 0.03 | -0.04 | 0.09 | 0.44 | 0.61 |
| *Note.* Condition was dummy-coded such that all comparisons above are in relation to the nonsocial list condition. | | | | | | | |

| **Table S11.** |  |  |  |  |  |  |  |
| --- | --- | --- | --- | --- | --- | --- | --- |
| *Residualized Change Models Comparing the Effects of the Social Letter vs. Social List Condition at Post-Test (T8) and Follow-Up (T9).* | | | | | | | |
| **Outcome** | ***b*** | ***SE*** | **Partial *r*** | **Partial *r* LLCI** | **Partial *r* ULCI** | ***p*** | ***p (adjusted)*** |
| Autonomy |  |  |  |  |  |  |  |
| Post-test | 0.10 | 0.14 | 0.02 | -0.04 | 0.09 | 0.47 | 0.63 |
| Follow-up | 0.00 | 0.14 | 0.00 | -0.06 | 0.06 | 0.99 | 1.00 |
| Competence |  |  |  |  |  |  |  |
| Post-test | 0.11 | 0.14 | 0.03 | -0.04 | 0.09 | 0.42 | 0.60 |
| Follow-up | -0.07 | 0.15 | -0.02 | -0.08 | 0.05 | 0.63 | 0.74 |
| Connectedness |  |  |  |  |  |  |  |
| Post-test | 0.24 | 0.13 | 0.06 | -0.01 | 0.12 | 0.08 | 0.17 |
| Follow-up | 0.29 | 0.15 | 0.06 | 0.00 | 0.13 | 0.05 | 0.11 |
| Elevation |  |  |  |  |  |  |  |
| Post-test | 0.72 | 0.15 | 0.16 | 0.10 | 0.22 | < .001 | < .001 |
| Follow-up | 0.42 | 0.15 | 0.09 | 0.02 | 0.15 | 0.01 | 0.02 |
| Gratitude |  |  |  |  |  |  |  |
| Post-test | 0.29 | 0.09 | 0.10 | 0.04 | 0.16 | 0.00 | 0.00 |
| Follow-up | 0.21 | 0.10 | 0.07 | 0.01 | 0.13 | 0.03 | 0.08 |
| Indebtedness |  |  |  |  |  |  |  |
| Post-test | 1.02 | 0.22 | 0.15 | 0.09 | 0.21 | < .001 | < .001 |
| Follow-up | 0.33 | 0.23 | 0.05 | -0.02 | 0.11 | 0.15 | 0.28 |
| Negative Affect |  |  |  |  |  |  |  |
| Post-test | 0.01 | 0.10 | 0.00 | -0.06 | 0.07 | 0.95 | 0.97 |
| Follow-up | 0.06 | 0.11 | 0.02 | -0.05 | 0.08 | 0.57 | 0.71 |
| Positive Affect |  |  |  |  |  |  |  |
| Post-test | 0.48 | 0.13 | 0.12 | 0.06 | 0.18 | < .001 | 0.00 |
| Follow-up | 0.37 | 0.14 | 0.08 | 0.02 | 0.15 | 0.01 | 0.02 |
| Life Satisfaction |  |  |  |  |  |  |  |
| Post-test | 0.27 | 0.11 | 0.08 | 0.02 | 0.14 | 0.01 | 0.03 |
| Follow-up | 0.23 | 0.12 | 0.06 | 0.00 | 0.13 | 0.05 | 0.11 |
| *Note.* Condition was dummy-coded such that all comparisons above are in relation to the social list condition. | | | | | | | |

| **Table S12.** |  |  |  |  |  |  |  |
| --- | --- | --- | --- | --- | --- | --- | --- |
| *Residualized Change Models Comparing the Effects of the Social Letter vs. the Unconstrained List Condition at Post-Test (T8) and Follow-Up (T9).* | | | | | | | |
| **Outcome** | ***b*** | ***SE*** | **Partial *r*** | **Partial *r* LLCI** | **Partial *r* ULCI** | ***p*** | ***p (adjusted)*** |
| Autonomy |  |  |  |  |  |  |  |
| Post-test | -0.01 | 0.13 | 0.00 | -0.07 | 0.06 | 0.93 | 0.96 |
| Follow-up | -0.06 | 0.14 | -0.01 | -0.08 | 0.05 | 0.68 | 0.78 |
| Competence |  |  |  |  |  |  |  |
| Post-test | -0.12 | 0.14 | -0.03 | -0.09 | 0.04 | 0.38 | 0.56 |
| Follow-up | -0.14 | 0.14 | -0.03 | -0.09 | 0.03 | 0.34 | 0.52 |
| Connectedness |  |  |  |  |  |  |  |
| Post-test | 0.15 | 0.13 | 0.04 | -0.03 | 0.10 | 0.25 | 0.42 |
| Follow-up | 0.08 | 0.14 | 0.02 | -0.05 | 0.08 | 0.57 | 0.71 |
| Elevation |  |  |  |  |  |  |  |
| Post-test | 0.42 | 0.14 | 0.10 | 0.03 | 0.16 | 0.00 | 0.01 |
| Follow-up | 0.21 | 0.15 | 0.05 | -0.02 | 0.11 | 0.16 | 0.30 |
| Gratitude |  |  |  |  |  |  |  |
| Post-test | 0.07 | 0.09 | 0.03 | -0.04 | 0.09 | 0.42 | 0.59 |
| Follow-up | 0.08 | 0.09 | 0.03 | -0.03 | 0.09 | 0.36 | 0.54 |
| Indebtedness |  |  |  |  |  |  |  |
| Post-test | 1.12 | 0.21 | 0.17 | 0.11 | 0.23 | < .001 | < .001 |
| Follow-up | 0.60 | 0.22 | 0.09 | 0.03 | 0.15 | 0.01 | 0.02 |
| Negative Affect |  |  |  |  |  |  |  |
| Post-test | -0.01 | 0.10 | 0.00 | -0.07 | 0.06 | 0.91 | 0.95 |
| Follow-up | -0.02 | 0.11 | 0.00 | -0.07 | 0.06 | 0.88 | 0.94 |
| Positive Affect |  |  |  |  |  |  |  |
| Post-test | 0.08 | 0.13 | 0.02 | -0.04 | 0.08 | 0.53 | 0.68 |
| Follow-up | 0.11 | 0.13 | 0.03 | -0.04 | 0.09 | 0.41 | 0.58 |
| Life Satisfaction |  |  |  |  |  |  |  |
| Post-test | -0.03 | 0.10 | -0.01 | -0.07 | 0.05 | 0.74 | 0.83 |
| Follow-up | -0.03 | 0.11 | -0.01 | -0.07 | 0.05 | 0.79 | 0.86 |
| *Note.* Condition was dummy-coded such that all comparisons above are in relation to the unconstrained list condition. | | | | | | | |

| **Table S13.** |  |  |  |  |  |  |  |
| --- | --- | --- | --- | --- | --- | --- | --- |
| *Residualized Change Models Comparing the Effects of the Social Letter vs. Other Gratitude Exercises at Post-Test (T8) and Follow-Up (T9).* | | | | | | | |
| **Outcome** | ***b*** | ***SE*** | **Partial *r*** | **Partial *r* LLCI** | **Partial *r* ULCI** | ***p*** | ***p (adjusted)*** |
| Autonomy |  |  |  |  |  |  |  |
| Post-test | 0.02 | 0.11 | 0.01 | -0.07 | 0.08 | 0.88 | 0.94 |
| Follow-up | -0.10 | 0.12 | -0.03 | -0.11 | 0.04 | 0.41 | 0.59 |
| Competence |  |  |  |  |  |  |  |
| Post-test | 0.08 | 0.12 | 0.03 | -0.05 | 0.1 | 0.51 | 0.66 |
| Follow-up | -0.11 | 0.12 | -0.04 | -0.11 | 0.04 | 0.37 | 0.54 |
| Connectedness |  |  |  |  |  |  |  |
| Post-test | 0.13 | 0.11 | 0.05 | -0.03 | 0.12 | 0.25 | 0.42 |
| Follow-up | 0.14 | 0.12 | 0.05 | -0.03 | 0.12 | 0.24 | 0.41 |
| Elevation |  |  |  |  |  |  |  |
| Post-test | 0.57 | 0.12 | 0.19 | 0.11 | 0.26 | < .001 | < .001 |
| Follow-up | 0.27 | 0.12 | 0.08 | 0.01 | 0.16 | 0.03 | 0.07 |
| Gratitude |  |  |  |  |  |  |  |
| Post-test | 0.21 | 0.07 | 0.11 | 0.03 | 0.18 | 0.01 | 0.01 |
| Follow-up | 0.15 | 0.08 | 0.08 | 0.00 | 0.15 | 0.05 | 0.12 |
| Indebtedness |  |  |  |  |  |  |  |
| Post-test | 0.98 | 0.18 | 0.21 | 0.13 | 0.28 | < .001 | < .001 |
| Follow-up | 0.40 | 0.19 | 0.08 | 0.01 | 0.16 | 0.03 | 0.08 |
| Negative Affect |  |  |  |  |  |  |  |
| Post-test | -0.03 | 0.08 | -0.01 | -0.09 | 0.06 | 0.72 | 0.81 |
| Follow-up | 0.00 | 0.09 | 0.00 | -0.08 | 0.08 | 0.98 | 0.99 |
| Positive Affect |  |  |  |  |  |  |  |
| Post-test | 0.34 | 0.11 | 0.12 | 0.04 | 0.2 | 0.00 | 0.01 |
| Follow-up | 0.21 | 0.12 | 0.07 | -0.01 | 0.15 | 0.07 | 0.15 |
| Life Satisfaction |  |  |  |  |  |  |  |
| Post-test | 0.13 | 0.09 | 0.06 | -0.02 | 0.13 | 0.13 | 0.26 |
| Follow-up | 0.15 | 0.10 | 0.06 | -0.02 | 0.14 | 0.12 | 0.25 |
| *Note.* Condition was dummy-coded such that all comparisons above are in relation to the list-writing conditions (social and nonsocial lists) and nonsocial letter condition. | | | | | | | |
